# Comparative Effects of Partial Body Weight-Supported and Loaded Treadmill Training on Motor Performance in Children with Cerebral Palsy: A Randomized Clinical Trial

**DOI:** 10.3390/medicina61071125

**Published:** 2025-06-22

**Authors:** Abdulmajeed Alotaibi, Alaa Ibrahim, Raafat Ahmed, Turki Abualait, Mohammed Jamal

**Affiliations:** 1 Department of Physical Therapy, Children’s Hospital, Ministry of Health, Taif 26514, Saudi Arabia; 2 Department of Physical Therapy, College of Applied Medical Sciences, Imam Abdulrahman Bin Faisal University, Dammam 34212, Saudi Arabia; aiibrahim@iau.edu.sa (A.I.); rmahmed@iau.edu.sa (R.A.); tsabualait@iau.edu.sa (T.A.); 3 Al Noor Specialist Hospital, Makkah 24241, Saudi Arabia; msjamal@moh.gov.sa

**Keywords:** cerebral palsy, gait, treadmill, muscle strength, postural control

## Abstract

*Background and Objectives:* Children with cerebral palsy (CP) improve walking abilities through partial body weight-supported treadmill training (PBWSTT) and loaded treadmill training (LTT), but there is no consensus on the most effective method. This study aimed to evaluate the effects of PBWSTT and LTT on spatiotemporal gait parameters in children with CP. *Materials and Methods:* A randomized clinical trial involved 25 children aged 12+ with spastic diplegic CP from various outpatient clinics in Taif and Makkah between January 2024 and January 2025. Participants were randomly assigned to PBWSTT (30% body weight support, *n* = 12) or LTT (60% lower limb weight loading, *n* = 13) with 45 min sessions three times per week for eight weeks, including conventional therapy. *Results:* The spatiotemporal gait parameters (such as gait speed, cadence, stride length, swing phase, and swing width) significantly improved within the PBWSTT and LTT groups, but no significant difference was found between the groups. The gross motor function measure, dimension E (for walking, running, and jumping), showed significantly higher improvement in the PBWSTT group compared to the LTT group (*p* = 0.047). *Conclusions:* This study indicates that PBWSTT and LTT can improve gait parameters in children with CP, with PBWSTT promoting postural control and LTT improving mobility. These findings suggest that the proposed rehabilitation strategies can significantly improve the functional outcomes of pediatric cerebral palsy patients.

## 1. Introduction

Cerebral palsy (CP) is the most common motor disability in childhood, resulting from a non-progressive injury to the developing brain. It often leads to impairments in muscle tone, posture, and motor function, with spastic diplegia being the most prevalent subtype affecting lower limb mobility and gait performance [1,2]. Gait abnormalities in children with CP significantly limit functional mobility, reduce independence in daily activities, and negatively impact quality of life and social participation [3,4]. Therefore, improving gait parameters is a major focus of pediatric neurorehabilitation.

Task-oriented training (TOT) is a widely used therapeutic approach that involves practicing context-specific motor tasks, such as walking, to promote functional recovery and neuroplasticity [5,6]. Within the TOT framework, two treadmill-based interventions—partial body weight-supported treadmill training (PBWSTT) and loaded treadmill training (LTT)—have gained clinical attention. PBWSTT uses a harness system to partially offload body weight, enabling repetitive, safe gait practice with reduced musculoskeletal strain [7]. In contrast, LTT applies additional external load, typically in the form of ankle weights, to the lower limbs to increase resistance and muscular effort during walking [8,9].

Numerous studies have demonstrated that PBWSTT can improve walking speed, endurance, and motor control in children with CP by facilitating early gait initiation and reducing the energy cost of ambulation [10,11]. Emerging evidence also supports the use of LTT for enhancing muscle strength, balance, and walking function [12]. However, the literature lacks consensus on which approach is superior, as both interventions have shown similar functional gains but rely on distinct physiological mechanisms—PBWSTT reduces the load to facilitate movement, while LTT increases resistance to challenge and strengthens lower limb muscles [13].

Given this gap, there is a need for a direct comparison of PBWSTT and LTT within a randomized controlled framework to determine their relative effectiveness in improving motor function and gait outcomes in children with spastic diplegic CP. This study aims to evaluate and compare the effects of PBWSTT and LTT using within- and between-group analyses, assessing their impact on gross motor function and walking ability. The findings will inform evidence-based clinical decision-making in pediatric rehabilitation.

## 2. Materials and Methods

### 2.1. Study Design and Setting

A randomized clinical trial with a parallel arm design was developed in line with the 2013 Declaration of Helsinki and the CONSORT statement for nonpharmacologic treatment interventions [14,15]. This study was conducted in outpatient physical therapy clinics at Children’s Hospital, in Taif and Alnoor Specialist Hospital in Makkah between January 2024 and January 2025. The trial aimed to assess the effectiveness of PBWSTT and LTT on spatiotemporal gait parameters in children with CP.

### 2.2. Study Participants and Eligibility Criteria

Children aged 12 years or younger with the diagnosis of spastic diplegic CP and a Gross Motor Function Classification System (GMFCS) level I–III, who can stand independently or with support and walk at least 20 m with or without walking aids, were eligible for study participation. The eligible children were recruited using a convenience sampling technique. Exclusion criteria were diagnosis of hemiplegic CP, presence of comorbidities, severe CP symptoms affecting daily activities, and severe cognitive or communicative impairments. Children classified as GMFCS levels IV and V were excluded due to the severity of motor impairments limiting treadmill participation. Additionally, children with severe cognitive impairment (IQ < 50, assessed by WISC-IV) or severe communication difficulties (CFCS levels IV and V) were excluded to ensure compliance with intervention protocols and safety during training.

### 2.3. Recruitment

Potential participants were identified and selected from children referred to outpatient physical therapy clinics. Families of children who satisfied the preliminary study requirements were approached and given thorough information about the program’s goals, methods, and possible advantages. Interested families were then contacted to take part in this study. A thorough screening process was carried out to ensure that the children were suitable. This method entailed analyzing the child’s medical history, assessing their present physical and cognitive ability, and determining whether they fit this study’s inclusion requirements. The screening was designed to establish whether the child was qualified for the program and could participate safely and efficiently.

### 2.4. Sample Size Calculation

The sample size was calculated to detect a 10% difference in Gross Motor Function Measure (GMFM) scores between the PBWSTT and LTT groups, assuming a large effect size (Cohen’s *d* = 0.8), a significance level (α) of 0.05, and 90% statistical power, while accounting for a 20% expected dropout rate [16]. Using G*Power software (version 3.1), the required sample was estimated to be 34 participants. Children with spastic diplegic cerebral palsy (*n* = 34) were then randomly assigned to the PBWSTT and LTT groups in equal numbers to ensure balanced allocation, minimize selection bias, and enhance the internal validity of this study. Although only 25 children completed the trial due to attrition, this study’s observed outcomes remained consistent with previous research [17,18], and the implications of the reduced sample size on statistical power and interpretation have been acknowledged and discussed accordingly.

### 2.5. Randomization, Allocation Concealment, and Blinding

To minimize selection bias, participants were randomly assigned to intervention groups using a computer-generated sequence prepared by an independent researcher, with group allocations concealed in sequentially numbered, opaque, sealed envelopes opened only after enrollment. While therapists could not be blinded due to the nature of the interventions, outcome assessors remained blinded throughout this study, and participants and their caregivers were not informed of group-specific hypotheses. To reduce performance bias related to therapist-child interaction, all therapists followed a standardized intervention protocol and received prior training to ensure consistent delivery of both PBWSTT and LTT interventions. Therapist assignments were balanced across groups to avoid differential influence.

### 2.6. Interventions

Children in the PBWSTT group received both conventional treatment and PBWSTT sessions at the rehabilitation center’s outpatient clinic. The PBWSTT procedure utilizes a motorized treadmill from a healthcare facility in China, equipped with an unweighting system from LiteGait^®^ Pediatric Mobility Research, Tempe, AZ, USA. PBWSTT sessions reduced 30% of children’s body weight, lasting 45 min, three times per week for eight weeks, using a treadmill speed of 0.1 KM/h [17].

Treadmill speed was increased incrementally by 0.1–0.2 km/h each session based on participant tolerance and gait quality. All sessions were conducted one-on-one under the supervision of a licensed pediatric physical therapist. Adherence was monitored via attendance logs, with an overall adherence rate of 92%, and no participants withdrew due to intervention-related issues.

The LTT group of children underwent both conventional treatment sessions and loaded treadmill training. LTT is a form of exercise where a child is placed on a motorized treadmill with an additional load attached to their lower extremities. Additional weight was added to the child’s lower limb, representing 60% of the child’s lower extremity weight [19]. The study by Robert K. Jensen was used to calculate the lower extremity weight of children [20]. Both procedures were introduced under the guidance of a pediatric physical therapist who has experience in treadmill settings.

Fifty minutes of treadmill training intervention timeline for both groups involved introducing the treadmill, explaining the process in a child-friendly manner, and encouraging questions or concerns within the first five minutes. For the following five minutes, the children were initially warmed up on a treadmill at a slow speed, gradually increasing the speed to a comfortable walking rate. Children participated in a 35 min treadmill training session, gradually increasing pace and receiving therapist assistance, while walking continuously in monitored form, posture, and energy levels. In the cooldown stage in the last five minutes, children gradually reduced treadmill speed, walked slowly or stepped off the treadmill, and performed light movements to relax muscles.

Both groups underwent identical conventional therapy sessions, focusing on interventions aimed at addressing motor function and physical impairments. The interventions involved motion exercises for joint flexibility, neurodevelopmental approaches for motor control, and impairment-based therapies like targeted stretching and strengthening exercises for muscle power and endurance. The conventional therapy sessions, lasting 30 min per session, were conducted three times weekly for eight weeks, ensuring equal baseline treatment for both groups.

### 2.7. Outcome Measures

The primary and secondary outcome measures were evaluated at both baseline and after eight weeks of sessions. The primary outcome measure consisted of quantitative spatiotemporal gait parameters captured using the Physilog^®^ 5 inertial measurement system (GaitUp S.A., Lausanne, Switzerland). This foot-worn platform integrates triaxial accelerometers (±3 g), gyroscopes (±800°/s), and barometric pressure sensors, sampling at 128 Hz to compute 3D foot kinematics during ambulation. Sensors were affixed bilaterally to participants’ footwear using low-profile mounts, enabling unobstructed gait analysis during both controlled walkway trials of the 20 m straight path. The system’s proprietary algorithms extracted several discrete gait metrics, including temporal parameters (stance/swing phase ratios, double-support duration), spatial characteristics (stride length, gait velocity), and foot strike/lift-off angles, with raw data processed through Gait Analyzer 5.2 software to minimize movement artifacts. Validation studies demonstrated robust psychometric properties in children with CP, with mean absolute errors of 3.4 ± 4.6 cm for stride length, 4.3 ± 4.2 cm/s for gait velocity, and intraclass correlation coefficients exceeding 0.90 for critical parameters [21].

Secondary outcome measures included the Gross Motor Function Measure-88 (GMFM-88), Pediatric Reach Test (PRT), Modified Timed Up and Go (mTUG), Five Times Sit-to-Stand Test (FTSST), Modified Ashworth Scale (MAS), and Hand-held dynamometer (HHD). The GMFM-88 is a standardized tool used to assess changes in gross motor function across two dimensions (D and E), using a 4-point ordinal scoring system to quantify functional capacity. The GMFM-D was utilized for measuring standing, while E was used for walking, running, or jumping. Administration follows a standardized protocol where trained assessors document the best of three performance trials per item, with verbal encouragement or demonstrations permitted. The GMFM-88 is a reliable and valid tool for measuring the GMFM in children with CP [22].

The PRT, a modified version of the Functional Reach Test, measures dynamic balance control in standing positions, focusing on forward reaching tasks for children. The PRT is a reliable and valid tool for children with CP, demonstrating good to excellent test–retest reliability [23]. The mTUG test was used to evaluate functional mobility and dynamic balance in children with CP, using standardized procedures in a controlled rehabilitation setting. Participants sat on a stable stool, adjusted for 90° hip and knee flexion, and were instructed to stand, walk 3 m to touch a visual target, turn around, and return to sit down. The mTUG, unlike traditional TUG, uses child-friendly instructions to improve comprehension in children with CP. Timing starts when standing and ends upon reseating, with three trials and no physical assistance provided. The mTUG is a reliable and accurate tool for measuring children with CP, with excellent test–retest reliability and minimal detectable change values, ranging from 1.40 to 8.74 s [24].

The FTSST evaluates functional lower limb strength, transitional mobility, and movement efficiency in children with CP, focusing on repeat sit-to-stand transitions for daily activities, such as rising from chairs or toilets. The test was conducted using a standardized chair height of 90° hip and knee flexion, without armrests or back support, and participants were to stand and sit down five times quickly upon the verbal cue “Go.” Timing commenced at the initiation of movement and concluded when the child touched the chair after the fifth repetition. Participants completed three trials after practice, with failures recorded as test failures. Contextualizing performance was observed using compensating techniques like upper limb support. The FTSST is highly reliable and precise for children with CP, with high test–retest reliability and minimal measurement error when using three trials [25].

The MAS scale assesses spasticity in children with CP, providing a standardized method for quantifying muscle tone during passive movement. Spasticity was assessed by moving the target joint through its range of motion at high velocity to elicit a stretch reflex. Each muscle group (e.g., hamstrings, gastrocnemius, hip adductors) was graded according to the MAS criteria: no increase in muscle tone, slight resistance at end-range, mild resistance through ≤50% of range, more marked resistance through most of range, considerable resistance, passive movement difficult, and rigid joint indicated by 0, 1, 1+, 2, 3, and 4, respectively. Testing was performed with the child relaxed, and three repeated measures were taken to improve reliability. The MAS has varying reliability in children with CP, ranging from poor to excellent, depending on the muscle group [26,27]. The Spasticity Index was determined by combining MAS scores for lower extremity muscle groups.

The HHD tool (Commander PowerTrack II, JTECH Medical, Midvale, UT, USA) quantifies strength in hip extensors, knee extensors, and ankle plantar flexors, providing crucial data for weight-bearing activities and functional mobility in children with CP. The highest value of the three trials in Newtons was recorded for analysis. The evaluated muscle strength was measured using standardized positions and stabilization methods [28].

### 2.8. Statistical Analysis

The Shapiro–Wilk test was used to determine the data distribution. Basic statistics were presented, like mean and standard deviation (SD) for continuous variables and count and percentage (%) for categorical variables. The Chi-square test for categorical variables determined the significant difference between the PBWSTT and LTT groups. The independent and paired *t*-tests were used for continuous variables to compare between and within groups, respectively. All statistical analyses were performed using IBM SPSS Statistics (Version 25; IBM Corp., Armonk, NY, USA), with a predetermined significance level of alpha = 0.05.

## 3. Results

**Figure 1:**
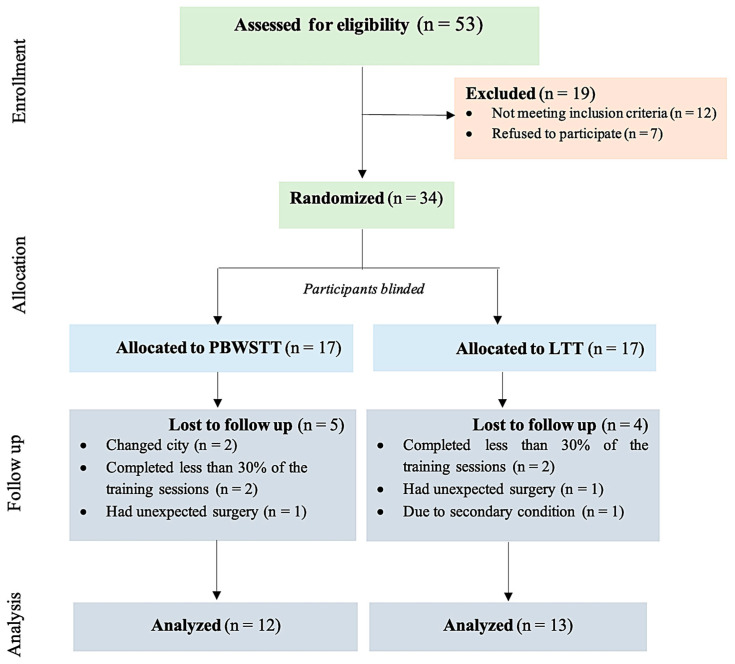
Flowchart of the study participants.

A total of 53 patients were examined successively, as they were visited at outpatient physical therapy clinics. Inclusion criteria were met by 34 of them. Out of the 19 patients who were not qualified for the program, 12 failed to meet the inclusion criteria and 7 refused to participate. Nine were lost in the follow-up due to relocation, less than 30% completion of a training session, and unexpected surgery (Figure 1). All 25 qualified patients took part in training sessions and completed the program. No undesirable medical events occurred during the program. All the subjects completed the final examination. Demographic and clinical characteristics are shown in Table 1.

Table 2 presents the primary and secondary outcomes within the two groups that were measured before and after interventions. Both groups showed significant improvement in GMFM-88 items, with the PBWSTT group showing increased GMFM-D (from 72.04 ± 33.62 to 75 ± 31.58, *p* = 0.004) and GMFM-E (from 67.24 ± 28.55 to 72.34 ± 29.03, *p* < 0.001) scores, indicating improved standing and walking-related functions. The LTT group showed significant improvements in GMFM-D from 56.2 ± 18.53 to 61.21 ± 16.92, *p* < 0.001, and E from 47 ± 19.46 to 51.62 ± 19.67, *p* < 0.001, with the PBWSTT group showing slightly more functional gains.

Functional mobility, as measured by the mTUG and FTSST, improved considerably in both groups. In the PBWSTT group, mTUG time fell from 11.35 ± 7.08 to 9.83 ± 7.15 s (*p* = 0.049), and FTSST time fell from 17.13 ± 12.68 to 13.68 ± 10.53 s (*p* = 0.007). Similarly, the LTT group showed a significant improvement in mTUG from 25.65 ± 39.68 to 16.53 ± 29.20 s (*p* = 0.021) and in FTSST from 20.95 ± 18.47 to 13.66 ± 14.81 s (*p* = 0.008). Despite extreme variability, particularly in the LTT group, the findings show increased transitional movements and dynamic balance following both interventions.

Gains in postural stability were noted on PRT, such that the PBWSTT group went up from 17.67 ± 4.50 to 22.67 ± 5.97 cm (*p* = 0.001), and the LTT group went from 13.77 ± 6.23 to 17.92 ± 6.20 cm (*p* < 0.001). These gains represent enhanced anticipatory postural stability and control that are important in daily functional tasks.

Notably, both PBWSTT and LTT yielded significant within-group improvements across a range of functional areas, including gross motor function, mobility, and balance. The findings show the potential of both treadmill interventions, with PBWSTT showing subtly more consistent improvement in standing function and postural control.

Table 3 presents in-group comparisons of lower limb muscle strength for both PBWSTT and LTT interventions. For the PBWSTT group, right hip extension strength was enhanced from a mean of 74.05 ± 37.42 to 105.11 ± 47.48 Newtons (*p* = 0.001) and left hip extension from 68.28 ± 32.93 to 102.90 ± 52.30 (*p* = 0.004). Right knee extension strength increased from 75.75 ± 30.86 to 95.30 ± 27.92, but not statistically significantly (*p* = 0.085). However, left knee extension improved considerably from 84.83 ± 25.62 to 101.85 ± 31.08 (*p* = 0.003). The PBWSTT group showed a significant increase in distal lower limb strength, with bilateral improvements in right (from 57.04 ± 25.74 to 99.31 ± 45.3, *p* < 0.001) and left ankle plantar flexors from 58.61 ± 25.95 to 102.15 ± 42.92, *p* = 0.001.

The LTT group also demonstrated parallel trends in strength improvement with notable increases in all the muscle groups tested. Right hip extension was elevated from 56.88 ± 33.74 to 74.37 ± 37.13 (*p* < 0.001) and left hip extension from 51.57 ± 30.62 to 75.83 ± 33.30 (*p* < 0.001). Knee extensor strength also increased significantly on both sides: right knee from 62.35 ± 27.76 to 79.12 ± 28.89 (*p* < 0.001) and left knee from 60.28 ± 30.11 to 75.90 ± 31.84 (*p* < 0.001).

The strength of ankle plantarflexion in the LTT group also showed significant gains. Right values rose from 47.97 ± 31.96 to 75.69 ± 40.58 (*p* < 0.001) and left values from 49.07 ± 29.89 to 75.83 ± 35.76 (*p* < 0.001). These findings imply that the inclusion of external limb loading while walking on a treadmill may have provided sufficient resistance to elicit adaptations in strength, especially in distal muscle groups. The Isometric Muscle Strength Index, combined scores of strengths of the lower limbs, were also significantly improved for the two groups. The PBWSTT group improved from 418.56 ± 140.99 to 606.61 ± 233.18 (*p* = 0.001) and the LTT group from 328.11 ± 174.56 to 456.75 ± 189.44 (*p* < 0.001).

Table A1 presents the spatiotemporal gait parameters within the groups. Walking speed increased significantly for both intervention groups. Mean speed increased in the PBWSTT group from 0.85 ± 0.23 to 0.97 ± 0.32 m/s (*p* = 0.046) and in the LTT group from 0.65 ± 0.41 to 0.83 ± 0.35 m/s (*p* = 0.014). Right and left cadence in the PBWSTT group increased from 118.95 ± 21.36 to 127.93 ± 16.41 (*p* = 0.004) and from 116.53 ± 20.77 to 126.86 ± 17.23 (*p* = 0.002) steps per minute, respectively. Similar trends were observed in the LTT group, with right cadence increasing from 101.04 ± 38.29 to 118.49 ± 32.64 (*p* = 0.028) and left cadence from 104.35 ± 31.94 to 115.06 ± 33.48 (*p* = 0.014). In the PBWSTT group, the right stride length increased from 0.82 ± 0.14 to 0.89 ± 0.14 m (*p* = 0.005), and the LTT group increased from 0.65 ± 0.33 to 0.75 ± 0.33 m (*p* = 0.020). Left stride length improved only in the LTT group, from 0.71 ± 0.29 to 0.82 ± 0.29 m (*p* = 0.015), while left stride length improvement in the PBWSTT group was not statistically significant (*p* = 0.145). The lift-off angle was significantly enhanced in both groups, PBWSTT (from 54.38 ± 14.38 to 61.43 ± 17.14, *p* = 0.002) and LTT (from 45.99 ± 17.52 to 56.84 ± 16.90, *p* = 0.021), suggesting enhanced propulsion and push-off capacity.

Table A2 presents the primary and secondary outcomes between the two groups that were measured before and after interventions. The GMFCS, a five-level ordinal scale measuring GMF, showed no significant differences before or after the intervention, possibly due to its short duration and gross nature. Spasticity Index scores were similar between groups at baseline and post-intervention, with LTT group showing less spasticity but not significantly different, suggesting no significant short-term effect on spastic muscle tone reduction. However, statistically significant differences in GMFM-88 dimension E were observed, with the PBWSTT group showing not significance after treatment compared to the LTT group (67.24 ± 28.55 vs. 46.99 ± 19.46; *p* = 0.48). The PBWSTT group showed higher post-intervention GMFM-88-dimension D and total scores than the LTT group, but not statistically significant (74.99 ± 31.58 versus 61.21 ± 16.92, *p* = 0.182). No significant difference in functional mobility was found between groups pre- or post-intervention, as measured by the mTUG and FTSST (PBWSTT: 9.83 ± 7.15 versus 13.68 ± 10.53 s, *p* = 0.448; LTT: 16.53 ± 29.20 versus 13.66 ± 14.81 s, *p* = 0.997). In addition, postural control was higher in the PBWSTT group after intervention compared to the LTT group, but not statistically significant (*p* = 0.064).

Table A3 presents between-group comparisons of lower limb muscle strength for both PBWSTT and LTT interventions. Before intervention, no significant differences were found in muscle strength measures, except for left knee extension strength, where the PBWSTT group had significantly greater force output than the LTT group (84.83 ± 25.62 N versus 60.28 ± 30.11 N, *p* = 0.039). The intervention significantly improved left knee extensor strength, with a significant trend towards significance, favoring the PBWSTT group over the LTT group (101.85 ± 31.08 N, versus 75.90 ± 31.84 N, *p* = 0.051).

Table A4 presents the spatiotemporal gait parameters within the groups. Walking speed increased significantly for both intervention groups. The PBWSTT group changed by 0.85 ± 0.23 m/s to 0.97 ± 0.32 m/s, whereas the LTT group changed by 0.65 ± 0.41 m/s to 0.83 ± 0.35 m/s, indicating significant improvements in gait efficiency. However, no significant differences were observed between the groups at pre- or post-intervention time points (*p* = 0.156 at pre and *p* = 0.326 at post). Cadence was enhanced in both groups, with PBWSTT showing larger post-intervention values. Right-side cadence enhanced from 118.95 ± 21.36 to 127.93 ± 16.41 in the PBWSTT group, a change from an enhancement from 101.04 ± 38.29 to 118.49 ± 32.64 in the LTT group. However, between-group differences were not statistically significant, with PBWSTT showing a significant improvement in right-side cadence, while LTT showed no significant improvement. Stride length was improved bilaterally in the PBWSTT group, with higher mean values than the LTT group, but these differences were not statistically significant. The post-intervention right stride length was 0.89 ± 0.14 m for PBWSTT and 0.75 ± 0.33 m for LTT (*p* = 0.194). Both interventions showed uniform stance and swing phase percentages, with right stance percentages in PBWSTT and LTT being similar post-intervention, and left stance percentages in both. There was a slight change in kinematic foot placement angles, including strike and lift-off angles, without group differences, with a slight decrease in right strike angle.

## 4. Discussion

The present study assessed the effects of PBWSTT and LTT on spatiotemporal gait parameters in children with CP. Both intervention groups demonstrated significant within-group improvements in primary gait outcomes, including gait speed, cadence, stride length, swing phase, and step width. However, no statistically significant differences were observed between the PBWSTT and LTT groups for these parameters. In contrast, the secondary outcome—GMFM dimension E, which assesses walking, running, and jumping—showed significantly greater improvement in the PBWSTT group compared to the LTT group, suggesting a potential advantage of PBWSTT in enhancing overall gross motor function.

Children with spastic diplegic CP often exhibit motor impairments such as selective motor control, muscle weakness, and gait pathology due to neuromuscular coordination issues [29,30]. Treadmill training enhances the activation of central pattern generators (CPGs) when combined with unloading or resistive-based strategies for repetitive task-specific movement [12]. This study found significant improvements in GMFM-88, TUG, FTSST, reach test, and muscle strength scores in the PBWSTT and LTT groups, suggesting weight-supported and resistance treadmill protocols can enhance gross motor function and neuromuscular capacity.

In this study, the GMFM-88 treadmill training significantly improved motor skill development and performance in children with CP, supporting previous research indicating its effectiveness [31,32]. Interestingly, this study found that PBWSTT showed better improvements in GMFM-88 dimension E compared to LTT, suggesting a more effective influence on higher-order dynamic motor functions like running and jumping. Similarly, the previous study indicates that unweighting in gait training reduces spastic muscle recruitment and improves motor patterning, enabling more complex movements [33].

PBWSTT is popular for task-specific gait training in decreased load conditions, improving postural alignment and coordination without falling risk, thereby enhancing stepping practice [33]. This study reveals that the PBWSTT group demonstrated greater improvement in GMFM-88 D pre- and post-intervention compared to the LTT group, indicating its effectiveness in enhancing postural stability and upright functional abilities. Chrysagis et al. (2012) found that children undergoing PBWSTT showed improved balance and vertical posture control compared to those receiving standard physiotherapy [31].

Both interventions improved gait speed, cadence, and stride length in children with CP, consistent with previous studies by Han & Yun [12]. PBWSTT likely contributed to children’s progress by enabling them to use gait with improved symmetry and reduced compensatory mechanisms [34], whereas LTT likely achieved this by increasing proprioceptive feedback and muscle resistance during stride [35].

Both interventions improved rhythm and pacing in children with cerebral palsy, with early timing adjustments and step coordination predicting normal gait normalization among children with CP [30,36], despite no significant differences in temporal gait parameters between groups. Moreover, both groups’ stride length, cadence, and gait speed significantly improved. GMFM-88 E (walking function) revealed stronger post-intervention improvement in the PBWSTT group, indicating a closer connection between unloaded gait practice and functional ambulation [32]. These results were in line with a 2010 study by Willoughby et al. that found that children who participated in eight weeks of PBWSTT showed notable increases in step symmetry, walking efficiency, and endurance [12].

This study found significant strength changes in the LTT group, especially for ankle plantar flexors and knee extensors, and higher correlations between strength gains and functional measures. Previous research has also shown that treadmill-based training interventions can improve dynamic balance [30,37]. Interestingly, LTT showed marginal improvement in FTSST, possibly due to enhanced strength training benefits from applied resistance [38]. Su et al. (2013) [39] demonstrated that LTT enhances neuromuscular efficiency, as evidenced by increased EMG activation and recruitment patterns of postural muscles. In addition, LTT can enhance eccentric control and proprioceptive loading, crucial for stabilizing joint movement and adjusting the musculoskeletal system to overground requirements [12,39].

Post-intervention PRT showed improvement in postural control and trunk stability in both groups, crucial for mobility in CP, which can be improved through weight-supported and resistance-based treadmill therapies [40]. Isometric strength testing revealed significant gains in hip and knee extensors and ankle plantar flexors, with a greater internal correlation between strength gains and functional performance [17,41]. PBWSTT enhances muscle strength through neuromuscular coordination and optimized movement patterns, aligning with previous research indicating it enhances gait smoothness and load transfer without raising peak torque [39].

The research highlights the significance of enhancing balance and dynamic control in pediatric CP, with the LTT group showing better correlations with GMFM scores. The resistance protocol may have boosted trunk coactivation or core strength, potentially improving overall coordination [40]. The improvements align with the idea that trunk control and lower limb strength are interdependent for achieving stable gait patterns [42]. PBWSTT and LTT have similar therapeutic effects, but their differences may be due to differences in neuromuscular pathways and adaptation mechanisms, with PBWSTT promoting motor patterning and neuroplasticity [43].

In this study, both interventions improved performance, with PBWSTT performing better for transitional and static tasks, and LTT showing higher correlation with dynamic strength tests and endurance-related tasks. Gunawan et al. (2022) [33] suggest that combining unloading and resistance training can enhance neuromuscular learning and strength development in children with GMFCS II–III levels. Studies also support treadmill training in children with CP rehabilitation, highlighting its effectiveness in improving gait velocity, balance, and motor function, despite potential accessibility issues with robotic gait trainers [37,44,45].

This study highlights the functional gains of low-cost, therapist-delivered treadmill training in chronic movement impairment, emphasizing its scalability and practicality in daily clinical practice [46,47]. The two therapies align with Hebbian-type plasticity, promoting sensorimotor reorganization through repetitive goal-directed movement from a neurophysiological perspective [41]. PBWSTT may improve walking patterns, with LTT solidifying motor outflow pathways. Future research may use functional MRI or TTS to compare brain activity changes and clinical improvement [48,49]. The neurophysiological effects of treadmill-based gait training interventions are rooted in motor learning and neuroplasticity mechanisms. PBWSTT supports high-repetition, low-impact stepping practice, which is critical in remapping cortical motor maps and eliciting spinal locomotor circuit reorganization [49]. In contrast to LTT, it engages more voluntary strength pathways, potentially stimulating corticomotor excitability and strengthening load-adaptive postural strategy [50,51].

### Study Limitations

This study provides valuable data on the comparative effects of PBWSTT and LTT on spastic diplegic children with CP, but it also has several limitations. First is this study’s small sample size. Although the sample size calculation indicated a required total of 34 participants, the final analyzed sample included only 25 due to participant attrition. This reduction may have affected the statistical power, potentially limiting the detection of smaller effect sizes. Nevertheless, the observed outcomes were consistent with prior studies and showed clinically meaningful differences [18], suggesting that the main conclusions remain valid. Second, the eight-week treadmill intervention showed functional and strength gains, but long-term effects of motor learning, muscle adaptation, and neurological remodeling were not assessed. Short duration may have compromised the sensitivity of measures. Future studies include follow-up assessments to evaluate the durability of intervention effects over time. Third, this study lacks follow-up measures to determine the persistence or long-term stability of observed gains in gait and functional motor gains in CP, limiting its effectiveness. Fourth, the treadmill design, while blinding outcome assessors, may lead to performance bias and unintended influence during training sessions, as subjective therapist–child interaction styles and motivation can affect engagement and performance. Fifth, this study’s external validity may be limited due to participants’ enrollment from a regional hospital, potentially affecting the wider population. Lastly, this study’s limitations, including sample size and duration, highlight the need for cautious interpretation and suggest areas for improvement in future research.

## 5. Conclusions

This study suggests that PBWSTT is better for patients with postural control deficits, asymmetrical gait patterns, and impaired standing or walking abilities, while LTT is better for generalized muscle weakness. These findings emphasize the significance of personalized rehabilitation planning, which involves selecting interventions based on a child’s specific functional impairments, age, and therapeutic objectives. Future research should explore long-term outcomes, the combined use of both interventions, and larger group replication to establish standardized protocols for treadmill training in pediatric CP populations.

## Figures and Tables

**Table 1:** Demographic and clinical characteristics of all children at baseline.

Characteristics	Total	PBWSTT	LTT	*p*
Number	25	12	13	
Age in years, Mean ± SD	8.72 ± 2.11	9.33 ± 2.17	8.15 ± 1.95	0.748 ^a^
Gender, *n* (%)				
Boys	16 (64)	8 (66.7)	8 (61.5)	0.794 ^b^
Girls	9 (36)	4 (33.3)	5 (38.5)	
Height in cm, Mean ± SD	121.08 ± 14.12	126.75 ± 12.54	115.85 ± 13.89	0.052 ^a^
Weight in Kg, Mean ± SD	25.74 ± 11.04	28.97 ± 13.02	22.76 ± 8.27	0.165 ^a^
BMI in Kg/m^2^, Mean ± SD	16.86 ± 3.63	17.29 ± 4.46	16.47 ± 2.79	0.583 ^a^
Lower extremity length in years, Mean ± SD	63.4 ± 9.90	66.5 ± 8.51	60.53 ± 10.54	0.135 ^a^
Pre-Spasticity Index, Mean ± SD	8.0 ± 6.08	6.83 ± 4.57	9.08 ± 7.23	0.368 ^a^
Hand Dominance, *n* (%)				
Right-handed	22 (88)	10 (83.3)	12 (92.3)	0.499 ^b^
Left-handed	3 (12)	2 (16.7)	1 (7.7)	
Type of Tonal Abnormality (Spasticity), *n* (%)	25 (100)	12 (100)	13 (100)	10.00 ^b^
Ambulation Capacity, *n* (%)				
Walks Independently	19 (76)	10 (83.3)	9 (69.2)	0.419 ^b^
Walks with Mobility Device	6 (24)	2 (16.7)	4 (30.8)	
No Walking	0 (0)	0 (0)	0 (0)	
Pre GMFCS, *n* (%)				
I	12 (48)	4 (33.3)	8 (69.2)	0.168 ^b^
II	10 (40)	6 (50.0)	4 (23.1)	
III	3 (12)	2 (16.7)	1 (7.7)	

Abbreviations: GMFCS: Gross Motor Functional Classification System; PBWSTT: partial body weight-supported treadmill; LTT: loaded treadmill training; ^a^ Independent *t*-test. ^b^ Chi-square test.

**Table 2:** MAS, GMFM-88, mTUG, and FTSST (within-group analysis).

Parameters		PBWSTT					LTT				
		Mean	SD	*p*	95% CI		Mean	SD	*p*	95% CI	
					L	U				L	U
Spasticity Index	Before	6.83	4.57	NC	NC	NC	9.08	7.23	0.082 ^a^	−0.07	0.99
	After	6.83	4.57				8.62	7.42			
GMFM-88 D	Before	72.04	33.62	0.004 ^a^	−4.78	−1.13	56.2	18.53	0.000 ^a^	−6.83	−3.18
	After	75	31.58				61.21	16.92			
GMFM-88 E	Before	67.24	28.55	0.000 ^a^	−7.02	−3.16	47	19.46	0.000 ^a^	−6.34	−2.91
	After	72.34	29.03				51.62	19.67			
GMFM-88	Before	139.29	61.09	0.000 ^a^	−10.9	−5.2	103.19	37.53	0.000 ^a^	−12.05	−7.22
	After	147.34	59.48				112.83	35.98			
mTUG	Before	11.35	7.08	0.049 ^a^	0.005	3.03	25.65	39.68	0.021 ^a^	1.61	16.62
	After	9.83	7.15				16.53	29.2			
FTSS	Before	17.13	12.68	0.007 ^a^	1.17	5.74	20.95	18.47	0.008 ^a^	2.24	12.35
	After	13.68	10.53				13.66	14.81			
PRT	Before	17.67	4.5	0.001 ^a^	−7.36	−2.64	13.77	6.23	0.000 ^a^	−5.07	−3.24
	After	22.67	5.97				17.92	6.2			

Abbreviations: PRT: Pediatric Reach Test; FTSS: Five Times Sit-to-Stand Test; mTUG: Modified Timed Up and Go Test; PBWSTT: partial body weight-supported treadmill; LTT: loaded treadmill training; CI: confidence interval; SD: standard deviation; L: Lower; U: Upper; NC: Not Computed because the standard error of the difference is 0. ^a^ Paired *t*-test.

**Table 3:** Muscle strength (within-group analysis).

Parameters		PBWSTT					LTT				
		Mean	SD	*p*	95% CI		Mean	SD	*p*	95% CI	
					L	U				L	U
Rt Hip Extension	Before	74.05	37.42	0.001 ^a^	−45.89	−16.23	56.88	33.74	0.000 ^a^	−25.43	−9.55
	After	105.11	47.48				74.37	37.13			
Lt Hip Extension	Before	68.28	32.93	0.004 ^a^	−55.63	−13.62	51.57	30.62	0.000 ^a^	−34.64	−13.88
	After	102.9	52.3				75.83	33.3			
Rt Knee Extension	Before	75.75	30.86	0.085 ^a^	−42.27	3.16	62.35	27.76	0.000 ^a^	−23.42	−10.13
	After	95.3	27.92				79.12	28.89			
Lt Knee Extension	Before	84.83	25.62	0.003 ^a^	−27.14	−6.9	60.28	30.11	0.000 ^a^	−20.91	−10.34
	After	101.85	31.08				75.9	31.84			
Rt Ankle Plantar flexion	Before	57.04	25.74	0.000 ^a^	−60.57	−23.96	47.97	31.96	0.000 ^a^	−39.82	−15.63
	After	99.31	45.3				75.69	40.58			
Lt Ankle Plantar flexion	Before	58.61	25.95	0.001 ^a^	−64.38	−22.71	49.07	29.89	0.000 ^a^	−35.63	−17.89
	After	102.15	42.92				75.83	35.76			
Isometric Muscle Strength Index	Before	418.56	140.99	0.001 ^a^	−279.09	−97.02	328.11	174.56	0.000 ^a^	−155.86	−101.41
	After	606.61	233.18				456.75	189.44			

Abbreviations: PBWSTT: partial body weight-supported treadmill training; LTT: loaded treadmill training; CI: confidence interval; L: Lower; U: Upper; SD: standard deviation; ^a^ Paired *t*-test.

## Data Availability

The data supporting the findings and analyzed during this study are available from the corresponding author upon reasonable request.

## Abbreviations

The following abbreviations are used in this manuscript:

CP	Cerebral palsy
TOT	Task-oriented training
PBWSTT	Partial body weight-supported treadmill training
LTT	Loaded treadmill training
GMGCS	Gross Motor Function Classification System
GMFM-88	Gross Motor Function Measure-88
PRT	Pediatric Reach Test
FTSST	Five Times Sit-to-Stand Test
HHD	Hand-held dynamometer
MAS	Modified Ashworth Scale
SD	Standard deviation

## Appendix A. Detailed Intervention Protocols

**Table A1:** Spatiotemporal gait parameters within the group.

Parameters		PBWSTT					LTT				
		Mean	SD	Sig.	95% CI		Mean	SD	*p*	95% CI	
					L	U				L	U
Speed	Before	0.85	0.23	0.046 ^a^	−0.23	−0.002	0.65	0.41	0.014 ^a^	−0.31	−0.04
	After	0.97	0.32				0.83	0.35			
Rt Cadence	Before	118.95	21.36	0.004 ^a^	−14.36	−3.59	101.04	38.29	0.028 ^a^	−32.72	−2.18
	After	127.93	16.41				118.49	32.64			
Lt Cadence	Before	116.53	20.77	0.002 ^a^	−15.85	−4.82	104.35	31.94	0.014 ^a^	−18.89	−2.54
	After	126.86	17.23				115.06	33.48			
Rt Stride Length	Before	0.82	0.14	0.005 ^a^	−0.11	−0.02	0.65	0.33	0.020 ^a^	−0.18	−0.02
	After	0.89	0.14				0.75	0.33			
Lt Stride Length	Before	0.83	0.2	0.145 ^a^	−0.1	0.02	0.71	0.29	0.015 ^a^	−0.21	−0.03
	After	0.87	0.16				0.82	0.29			
Rt Stance Percentage	Before	62.65	5.12	0.386 ^a^	−1.69	4.03	65.15	5.43	0.116 ^a^	−0.8	6.35
	After	61.48	3.99				62.38	5.87			
Lt Stance Percentage	Before	64.11	4.04	0.104 ^a^	−0.49	4.52	62.19	7.05	0.743 ^a^	−3.44	2.52
	After	62.1	3.32				62.65	5.05			
Rt Swing Percentage	Before	37.35	5.12	0.699 ^a^	−2.76	1.92	34.83	5.44	0.118 ^a^	−6.35	0.82
	After	37.77	3.73				37.6	5.86			
Lt Swing Percentage	Before	35.89	4.04	0.104 ^a^	−4.52	0.49	37.88	7.06	0.669 ^a^	−2.43	3.65
	After	37.9	3.32				37.27	4.99			
Double Support	Before	26.19	7.74	0.158 ^a^	−1.12	6.07	26.82	11.93	0.641 ^a^	−3.76	5.88
	After	23.71	6.44				25.76	12.4			
Rt Strike Angle	Before	15.74	7.45	0.464 ^a^	−1.98	4.06	13.16	9.97	0.317 ^a^	−2.7	7.65
	After	14.69	7.44				10.69	8.01			
Lt Strike Angle	Before	16.3	6.98	0.503 ^a^	−2.13	4.09	13.28	11	0.960 ^a^	−4.09	3.9
	After	15.33	6.69				13.37	10.11			
Rt Lift-Off Angle	Before	54.38	14.38	0.002 ^a^	−10.89	−3.22	45.99	17.52	0.021 ^a^	−19.78	−1.92
	After	61.43	17.14				56.84	16.9			
Lt Lift-Off Angle	Before	53.92	12.65	0.076 ^a^	−13.28	0.78	52.2	15.15	0.003 ^a^	−14.93	−3.74
	After	60.17	18.36				61.54	15.56			
Rt Swing Width	Before	−0.03	0.02	0.504 ^a^	−0.02	0.01	−0.03	0.04	0.240 ^a^	−0.01	0.02
	After	−0.03	0.02				−0.04	0.02			
Lt Swing Width	Before	0.03	0.04	0.366 ^a^	−0.19	0.08	0.03	0.03	0.352 ^a^	−0.02	0.01
	After	0.08	0.23				0.03	0.03			

Abbreviations: PBWT: partial body weight-supported treadmill; LTT: loaded treadmill training; CI: confidence interval; L: Lower; U: Upper; SD: standard deviation; ^a^ Paired *t*-test.

**Table A2:** GMFCS, MAS, GMFM-88, mTUG, and FTSST (between-group analysis).

Parameters		Before Treatment					After Treatment				
		Mean	SD	*p*	95% CI		Mean	SD	*p*	95% CI	
					L	U				L	U
GMFCS	PBWSTT	14.92 (Mean rank)		0.168 ^b^	-	-	15.33 (Mean rank)		0.091 ^b^	-	-
	LTT	11.23 (Mean rank)			-	-	10.85 (Mean rank)			-	-
Spasticity Index	PBWSTT	6.83	4.57	0.368 ^a^	−7.3	2.81	6.83	4.57	0.482 ^a^	−6.94	3.37
	LTT	9.08	7.23				8.62	7.42			
GMFM-88 D	PBWSTT	72.04	33.62	0.154 ^a^	−6.37	38.06	75	31.58	0.182 ^a^	−6.93	34.52
	LTT	56.2	18.53				61.21	16.92			
GMFM-88 E	PBWSTT	67.24	28.55	0.048 ^a^	0.18	40.32	72.34	29.03	0.047 ^a^	0.35	41.08
	LTT	47	19.46				51.62	19.67			
GMFM-88	PBWSTT	139.29	61.09	0.086 ^a^	−5.48	77.66	147.34	59.48	0.090 ^a^	−5.79	74.8
	LTT	103.19	37.53				112.83	35.98			
mTUG	PBWSTT	11.35	7.08	0.232 ^a^	−38.38	9.78	9.83	7.15	0.448 ^a^	−24.64	11.24
	LTT	25.65	39.68				16.53	29.2			
FTSS	PBWSTT	17.13	12.68	0.556 ^a^	−17.04	9.4	13.68	10.53	0.997 ^a^	−10.7	10.74
	LTT	20.95	18.47				13.66	14.81			
PRT	PBWSTT	17.67	4.5	0.088 ^a^	−0.64	8.43	22.67	5.97	0.064 ^a^	−0.3	9.79
	LTT	13.77	6.23				17.92	6.2			

Abbreviations: PRT: Pediatric Reach Test; FTSS: Five Times Sit-to-Stand Test; mTUG: Modified Timed Up and Go Test; PBWSTT: partial body weight-supported treadmill; LTT: loaded treadmill training; CI: confidence interval; L: Lower; U: Upper; SD: standard deviation; NC: Not Computed because the standard error of the difference is 0. ^a^ Independent *t*-test; ^b^ Mann–Whitney test.

**Table A3:** Muscle strength (between-group analysis).

Parameters		Before Treatment					After Treatment				
		Mean	SD	*p*	95% CI		Mean	SD	*p*	95% CI	
					L	U				L	U
Rt Hip Exten	PBWSTT	74.05	37.42	0.240 ^a^	−12.26	46.61	105.11	47.48	0.083 ^a^	−4.37	65.84
	LTT	56.88	33.74				74.37	37.13			
Lt Hip Exten	PBWSTT	68.28	32.93	0.202 ^a^	−9.58	42.99	102.9	52.3	0.133 ^a^	−8.91	63.04
	LTT	51.57	30.62				75.83	33.3			
Rt Knee Exten	PBWSTT	75.75	30.86	0.265 ^a^	−10.85	37.65	95.3	27.92	0.169 ^a^	−7.36	39.72
	LTT	62.35	27.76				79.12	28.89			
Lt Knee Exten	PBWSTT	84.83	25.62	0.039 ^a^	1.32	47.79	101.85	31.08	0.051 ^a^	−0.11	52.02
	LTT	60.28	30.11				75.9	31.84			
Rt Ankle Plantar	PBWSTT	57.04	25.74	0.445 ^a^	−15.07	33.21	99.31	45.3	0.182 ^a^	−11.92	59.14
	LTT	47.97	31.96				75.69	40.58			
Lt Ankle Plantar	PBWSTT	58.61	25.95	0.405 ^a^	−13.71	32.79	102.15	42.92	0.108 ^a^	−6.27	58.91
	LTT	49.07	29.89				75.83	35.76			
Isometric Muscle Strength Index	PBWSTT	418.56	140.99	0.170 ^a^	−41.55	222.44	606.61	233.18	0.090 ^a^	−25.27	325.01
	LTT	328.11	174.56				456.75	189.44			

Abbreviations: PBWSTT: partial body weight-supported treadmill training; LTT: loaded treadmill training; CI: confidence interval; L: Lower; U: Upper; SD: standard deviation; ^a^ Independent *t*-test.

**Table A4:** Spatiotemporal gait parameters (between-group analysis).

Parameters		Before Treatment					After Treatment				
		Mean	SD	*p*	95% CI		Mean	SD	*p*	95% CI	
					L	U				L	U
Speed	PBWSTT	0.85	0.23	0.156 ^a^	−0.08	0.48	0.97	0.32	0.326 ^a^	−0.14	0.42
	LTT	0.65	0.41				0.83	0.35			
Rt Cadence	PBWSTT	118.95	21.36	0.167 ^a^	−8.06	43.87	127.93	16.41	0.377 ^a^	−12.24	31.1
	LTT	101.04	38.29				118.49	32.64			
Lt Cadence	PBWSTT	116.53	20.77	0.275 ^a^	−10.33	34.69	126.86	17.23	0.286 ^a^	−10.52	34.12
	LTT	104.35	31.94				115.06	33.48			
Rt Stride Length	PBWSTT	0.82	0.14	0.123 ^a^	−0.05	0.38	0.89	0.14	0.194 ^a^	−0.08	0.35
	LTT	0.65	0.33				0.75	0.33			
Lt Stride Length	PBWSTT	0.83	0.2	0.228 ^a^	−0.08	0.33	0.87	0.16	0.603 ^a^	−0.15	0.25
	LTT	0.71	0.29				0.82	0.29			
Rt Stance Percentage	PBWSTT	62.65	5.12	0.249 ^a^	−6.88	1.87	61.48	3.99	0.660 ^a^	−5.09	3.28
	LTT	65.15	5.43				62.38	5.87			
Lt Stance Percentage	PBWSTT	64.11	4.04	0.417 ^a^	−2.89	6.73	62.1	3.32	0.751 ^a^	−4.12	3.01
	LTT	62.19	7.05				62.65	5.05			
Rt Swing Percentage	PBWSTT	37.35	5.12	0.247 ^a^	−1.87	6.9	37.77	3.73	0.932 ^a^	−3.93	4.28
	LTT	34.83	5.44				37.6	5.86			
Lt Swing Percentage	PBWSTT	35.89	4.04	0.399 ^a^	−6.81	2.81	37.9	3.32	0.716 ^a^	−2.91	4.17
	LTT	37.88	7.06				37.27	4.99			
Double Support	PBWSTT	26.19	7.74	0.878 ^a^	−9.03	7.77	23.71	6.44	0.613 ^a^	−10.34	6.23
	LTT	26.82	11.93				25.76	12.4			
Rt Strike Angle	PBWSTT	15.74	7.45	0.475 ^a^	−4.76	9.91	14.69	7.44	0.209 ^a^	−2.4	10.42
	LTT	13.16	9.97				10.69	8.01			
Lt Strike Angle	PBWSTT	16.3	6.98	0.425 ^a^	−4.67	10.72	15.33	6.69	0.578 ^a^	−5.21	9.11
	LTT	13.28	11				13.37	10.11			
Rt Lift-Off Angle	PBWSTT	54.38	14.38	0.206 ^a^	−4.94	21.72	61.43	17.14	0.507 ^a^	−9.5	18.68
	LTT	45.99	17.52				56.84	16.9			
Lt Lift-Off Angle	PBWSTT	53.92	12.65	0.762 ^a^	−9.89	13.32	60.17	18.36	0.842 ^a^	−15.41	12.67
	LTT	52.2	15.15				61.54	15.56			
Rt Swing Width	PBWSTT	−0.03	0.02	0.730 ^a^	−0.03	0.02	−0.03	0.02	0.343 ^a^	−0.01	0.03
	LTT	−0.03	0.04				−0.04	0.02			
Lt Swing Width	PBWSTT	0.03	0.04	0.933 ^a^	−0.03	0.03	0.08	0.23	0.462 ^a^	−0.08	0.18
	LTT	0.03	0.03				0.03	0.03			

Abbreviations: PBWT: partial body weight-supported treadmill; LTT: loaded treadmill training; CI: confidence interval; L: Lower; U: Upper; SD: standard deviation; ^a^ Independent t test.
